# Around the Hospitals

**Published:** 1893-03-11

**Authors:** 


### AROUND THE HOSPITALS.

**The Royal National Hospital for Consumption,** Ventnor, of which Lord Rosebery is President, appeals particularly to national support, alleviating as it does the sufferers from this terribly prevalent disease. It is pleasant to record that the announcement was made at the recently-held annual meeting that the annual subscriptions for the past year show a decided increase. The bequests, amounting to £4,468, have also been exceptionally large. Donations, on the other hand, show a falling off. The expenditure of such an institution as that at Ventnor, where the treatment, necessarily expensive, is most admirably carried out, must always be very large, and there is consequently pressing need for the funds for which the committee plead.

**The Middlesex Hospital,** it will be remembered, was forced to close three wards (surgical) for the entire re-flooring during the past summer. This has been satisfactorily accomplished, and improved fire-places have been added, and the new children's wards have been completed. Other improvements have also taken place in the hospital, all of which were very necessary. It was originally intended to close the whole hospital for a period during repairs, but as two other hospitals were closing at the same time, the Middlesex Hospital showed a commendable public spirit, and remained open at much inconvenience to itself. The number of in-patients was only reduced by 106 during the year, whilst the number of out-patients show a considerable increase. Some handsome donations have added to the funds of the institution. Annual subscriptions have risen, and contributions to the alms-boxes have also been larger during the past year. We note that the electric lighting has largely increased the expenditure under light and heating. Statistics on this point are of value, as the question of cost on this head is one that constantly arises in the construction of new buildings.

**The London Fever Hospital,** in Liverpool Road, held an annual meeting of governors on the 10th inst. Lord Balfour of Burleigh in the chair. Throughout the past year scarlet fever and measles having been especially prevalent, the resources of the institution were a good deal taxed. It is worthy of note that the mortality from scarlet fever was exceptionally low, being only 1·9 per cent. The much higher death rate amongst the sufferers from measles, reaching 8·1 per cent., will doubtless appear especially surprising to those who have been in the habit of regarding measles as a comparatively insignificant disease.

**The Cancer Hospital,** Brompton Road, held its forty-second annual meeting on the 22nd ult., Sir George S. Meason, J.P., occupying the chair. 811 in-patients received treatment during the year, and the number of attendances on the part of out-patients exceeds that of any previous year. Considerable additions and improvements have been made to the hospital, including the erection of a chapel and extra sleeping accommodation for the nurses. The reliable income of the institution is far less than the ordinary expenditure, the Committee therefore make an earnest appeal for funds to carry on the work of the institution.

